# Wideband Terminal Antenna System Based on Babinet’s Principle for Sub-6 GHz and Wi-Fi 6E/7 Applications

**DOI:** 10.3390/mi15060705

**Published:** 2024-05-26

**Authors:** Chong-Zhi Han, Guji Gong, Yan Wang, Jie Guo, Liang Zhang

**Affiliations:** 1 School of Ocean Information Engineering, Jimei University, Xiamen 361021, China; chongzhi_han@foxmail.com (C.-Z.H.); gongguji@163.com (G.G.); 2 Shenyang Aircraft Design and Research Institute, Shenyang 110035, China; sy601wangyan@163.com; 3 The Key Laboratory of Dependable Service Computing in Cyber Physical Society Ministry of Education, College of Microelectronics and Communication Engineering, Chongqing University, Chongqing 400044, China

**Keywords:** broadband antenna, impedance property analysis methodology, MIMO antenna system

## Abstract

In this paper, a novel input impedance analysis methodology based on Babinet’s principle to broaden bandwidth is proposed, and a broadband multiple-input and multiple-output (MIMO) antenna system is designed, fabricated, and measured for fifth-generation (5G) and Wireless Fidelity (Wi-Fi) 6E/7 mobile applications. By analyzing the input impedance of open-slot antennas and planar monopole antennas using numerical calculations, the characteristics of the input impedance can be obtained. We find that combining the two antenna types in parallel can significantly enhance the bandwidth. Then, the four-dimensional image calculated by MATLAB based on the parallel impedance formula is processed to validate the methodology. Thus, a broad antenna element based on the impedance property analysis methodology is achieved, which operates ranging from 2.6 GHz to 7.46 GHz. Moreover, the equivalent circuit of the antenna element is established to further verify the validity of the methodology. Finally, a broadband MIMO antenna system consisting of eight antenna elements is designed, fabricated, and measured, and the isolation performance is better than 12 dB. Acceptable total efficiency higher than 45% is also obtained with envelope correlation coefficients (ECCs) lower than 0.05. The proposed impedance property analysis methodology innovatively proposes a new way to increase bandwidth, which can be widely applied in various antenna designs. Also, reasonable results show that the proposed MIMO antenna system is a good candidate for 5G and Wi-Fi 6E/7 mobile applications.

## 1. Introduction

With the rapid development of communication technology, the growing demand for mobile terminals with both outdoor cellular and indoor Wireless Local Area Network (WLAN) communication capabilities is inevitable. Standards for the 5G of mobile communications have been released by the 3rd-Generation Partnership Project (3 GPP). Operating frequency band Sub-6 GHz includes n77 (3300–4200 MHz), n78 (3300–3800 MHz), and n79 (4400–5000 MHz). Furthermore, Wi-Fi protocol standards typically cover the 2.45 GHz (2400–2484 MHz) and 5 GHz (5150–5830 MHz) bands. In addition, the latest standard for WLAN communications divides the 6 GHz band (5925–7125 MHz) into Wi-Fi 6E/7. Hence, an antenna covering both Sub-6 GHz bands and Wi-Fi 6E/7 bands might be a promising candidate for future terminal devices. Some studies devoted to broadband antennas for 5G Sub-6 GHz have been published [1,2,3,4,5,6,7]. Also, several works have been reported to cover the Wi-Fi 6E frequency band for WLAN applications [8,9,10,11]. However, designing a broadband antenna meeting the needs of WLAN (2.45 GHz, 5 GHz and 6 GHz) and Sub-6 GHz bands is still a challenge. Ref. [12] reported a frequency reconfigurable antenna covering WLAN (2.45 GHz) and Sub-6 GHz (3.50 GHz), but it cannot operate at 6 GHz bands. Ref. [13] proposed a multiband antenna for 5G and Wi-Fi 6 applications; however, it cannot cover the entire Sub-6 GHz, and its operating frequency bands are not continuous. In [14], a compact 2 × 2 antenna array covering 5G and Wi-Fi bands (4.41–5.85 GHz) is presented, but it does work for Wi-Fi 6E/7 applications.

To address the bandwidth limitation of the MIMO antenna system for size-limited terminal equipment, various broadband techniques have been reported [15,16,17]. In [15], a dual-band antenna was obtained by embedding branch-line slits. Ref. [16] proposed a modified planar inverted F antenna (PIFA) with a parasitic patch. A novel center-fed wideband antenna was also proposed using the multi-mode resonance concept in [17]. However, the abovementioned methods cannot be utilized to design a wideband antenna covering 5G and Wi-Fi 6E/7 bands simultaneously.

In this paper, a novel impedance property analysis methodology based on Babinet’s principle to widen bandwidth is proposed. Then, an antenna element consisting of a planar monopole antenna and an open-slot antenna in parallel based on the impedance property analysis methodology is designed. Furthermore, a wideband MIMO antenna system composed of eight elements is presented, fabricated, and measured, operating in a range from 2.6 GHz to 7.46 GHz. Its isolation performance is better than 12 dB. A total efficiency higher than 45% has been achieved with ECCs lower than 0.05. The proposed methodology will propose a new way to design a broadband antenna system for 5G and Wi-Fi 6E/7 mobile terminals.

## 2. Design of the Antenna Element and MIMO Antenna System

**Figure 1:**
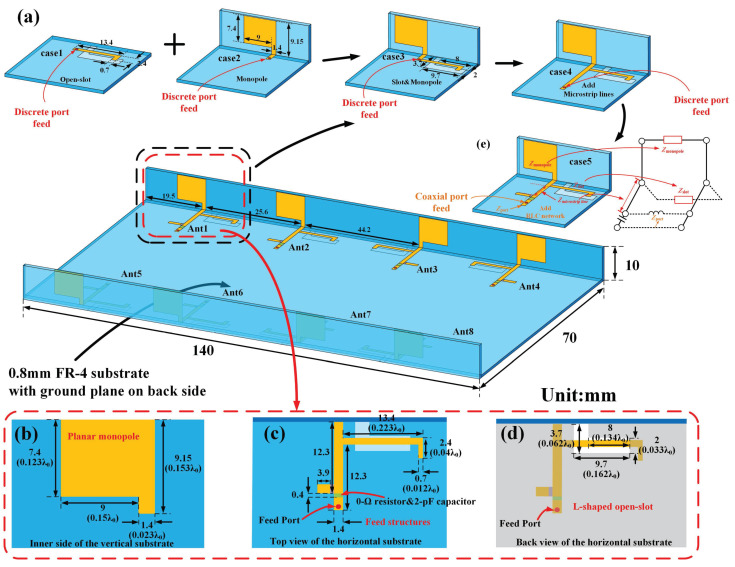
The evolution of the proposed antenna element and overall configuration of the MIMO antenna system: (**a**) The evolution of the proposed antenna element; The detailed dimensions of (**b**) planar monopole, (**c**) feed structures and (**d**) L-shaped open-slot; (**e**) The final configuration of the proposed antenna element and its equivalent circuit.

Figure 1 depicts the geometry of the proposed antenna element as well as the arrangements of the 8MIMO antenna system. The metal ground plane with a thickness of 0.035 mm is printed on the back of the 0.8-mm-thick horizontal FR-4 substrate ($\varepsilon_{r}$ = 4.6, tanδ = 0.025), the dimension of which is 140 × 70 ${\mathrm{mm}}^{2}$. Two vertical FR-4 substrates with a height of 10 mm represent the longer side frame of the smartphone. In addition, the detailed parameters of the antenna element consisting of the planar monopole antenna, the L-shaped open-slot antenna, and the feed structures are given in Figure 1b, Figure 1c, and Figure 1d, respectively. Also, the detailed dimensions of the main radiators in the wavelength $\lambda_{0}$ are given, where the wavelength $\lambda_{0}$ is at the center frequency. The planar monopole antenna is etched on the inner side of the longer vertical side frame, with a dimension of 7.4 × 10.4 ${\mathrm{mm}}^{2}$. The open-slot antenna is etched onto the bottom layer of the ground plane, which is fed by the L-shaped metallic branch on the top layer of the ground plane. The feed structure is composed of the microstrip line and the RLC network with a 0-Ω resistor and a 2-pF capacitor in series. The 0-Ω resistor position is reserved for impedance matching due to manufacturing error. The feed structure is covered on the top layer of the substrate and connected by a simple 50-Ω coaxial line. The eight abovementioned antenna elements are arranged symmetrically along two longer side frames of the terminal to obtain an eight-element MIMO antenna system for 5G and Wi-Fi 6E/7 mobile applications.

## 3. The Wideband Principle of the Proposed Antenna System

**Figure 2:**
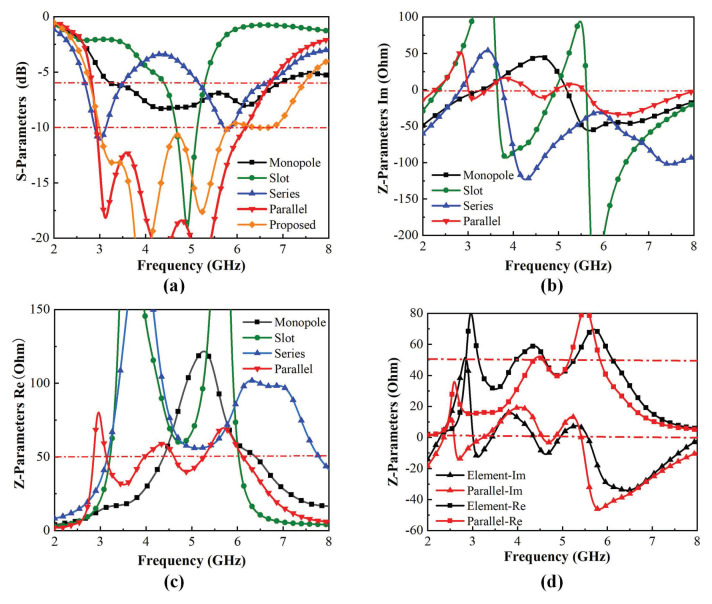
Simulated *S*-parameters and *Z*-parameters of the antennas in evolution: (**a**) Simulated *S*-parameters of monopole, slot, series-case, parallel-case and proposed antenna element; (**b**) The imaginary parts of the impedance of the monopole, slot, series-case and parallel-case; (**c**) The real parts of the impedance of the monopole, slot, series-case and parallel-case; (**d**) The real and imaginary parts of the impedance of the parallel-case and antenna element.

Figure 1a shows evolution of the proposed antenna element. Case 1 is the open-slot antenna, which operates from 4.5 GHz to 5.3 GHz, and case 2 is the planar monopole antenna, which covers the 3.5–6.6 GHz frequency, as illustrated in Figure 2a. Both open-slot antennas and planar monopole antennas cannot cover the frequency band of 5G (3300–5000 MHz) and Wi-Fi 6E/7 (5925–7125 MHz). Considering that the planar monopole antenna and open-slot antenna can be deemed as complementary counterparts approximatively, Babinet’s principle can be applied to improve the bandwidth [18,19]. Babinet’s principle and its extension show that if two antennas are complementary, their *Z*-parameters can be demonstrated by Equation (1). Figure 2b depicts the imaginary part of the impedance of the open-slot antenna and planar monopole antenna. It can be found that their impedance imaginary parts are opposite at around 3.7 GHz, which means that the impedance of the open-slot antenna is inductive and the impedance of the planar monopole antenna is capacitive when the frequency is less than 3.7 GHz, and the opposite is true when the frequency is larger than 3.7 GH, so they could be combined to widen the input impedance bandwidth. To validate whether a series or parallel connection is used to combine them, relevant simulations are carried out. Equations (2) and (3) represent the impedance of the open-slot antenna and planar monopole antenna, respectively. The parallel impedance $Z_{\mathrm{parallel}}$ and series impedance $Z_{\mathrm{series}}$ of $Z_{\mathrm{slot}}$ and $Z_{\mathrm{monopole}}$ can be calculated by Equations (4) and (5), as depicted in Figure 2. It can be found that the partial impedance curve of an open-slot antenna much greater than 50 Ω becomes closer to 50 Ω in Figure 2c in the parallel case. The partial impedance curve of an open-slot antenna much greater than 0 Ω becomes closer to 0 Ω in Figure 2b in the parallel case. The phenomenon indicates that a better match can be obtained in the parallel case. Thus, an open-slot antenna and planar monopole antenna are combined in parallel in case 3 in Figure 1a. Due to the change in feed structure after parallel, the microstrip line and the RLC network are added to tune the match in case 5 in Figure 1a.

(1) $$\begin{array}{c} Z_{m}Z_{s}=\frac{{Z_{0}}^{2}}{4}, \end{array}$$

(2) $$Z_{\mathrm{slot}}=R_{\mathrm{slot}}+j\cdot X_{\mathrm{slot}},$$

(3) $$Z_{\mathrm{monopole}}=R_{\mathrm{monopole}}+j\cdot X_{\mathrm{monopole}},$$

(4) $$\begin{array}{cc} Z_{\mathrm{parallel}} & =\frac{{R_{\mathrm{slot}}}^{2}R_{\mathrm{monopole}}+R_{\mathrm{slot}}{R_{\mathrm{monopole}}}^{2}+R_{\mathrm{slot}}{X_{\mathrm{monopole}}}^{2}+{X_{\mathrm{slot}}}^{2}R_{\mathrm{monopole}}}{{\left(R_{\mathrm{slot}}+R_{\mathrm{monopole}}\right)}^{2}+{\left(X_{\mathrm{slot}}+X_{\mathrm{monopole}}\right)}^{2}}\\ \; & +\;j\cdot \frac{{R_{\mathrm{slot}}}^{2}X_{\mathrm{monopole}}+X_{\mathrm{slot}}{R_{\mathrm{monopole}}}^{2}+{X_{\mathrm{slot}}}^{2}X_{\mathrm{monopole}}+X_{\mathrm{slot}}{X_{\mathrm{monopole}}}^{2}}{{\left(R_{\mathrm{slot}}+R_{\mathrm{monopole}}\right)}^{2}+{\left(X_{\mathrm{slot}}+X_{\mathrm{monopole}}\right)}^{2}}, \end{array}$$

(5) $$Z_{\mathrm{series}}=\left(R_{\mathrm{slot}}+R_{\mathrm{monopole}}\right)+j\cdot \left(X_{\mathrm{slot}}+X_{\mathrm{monopole}}\right),$$

**Figure 3:**
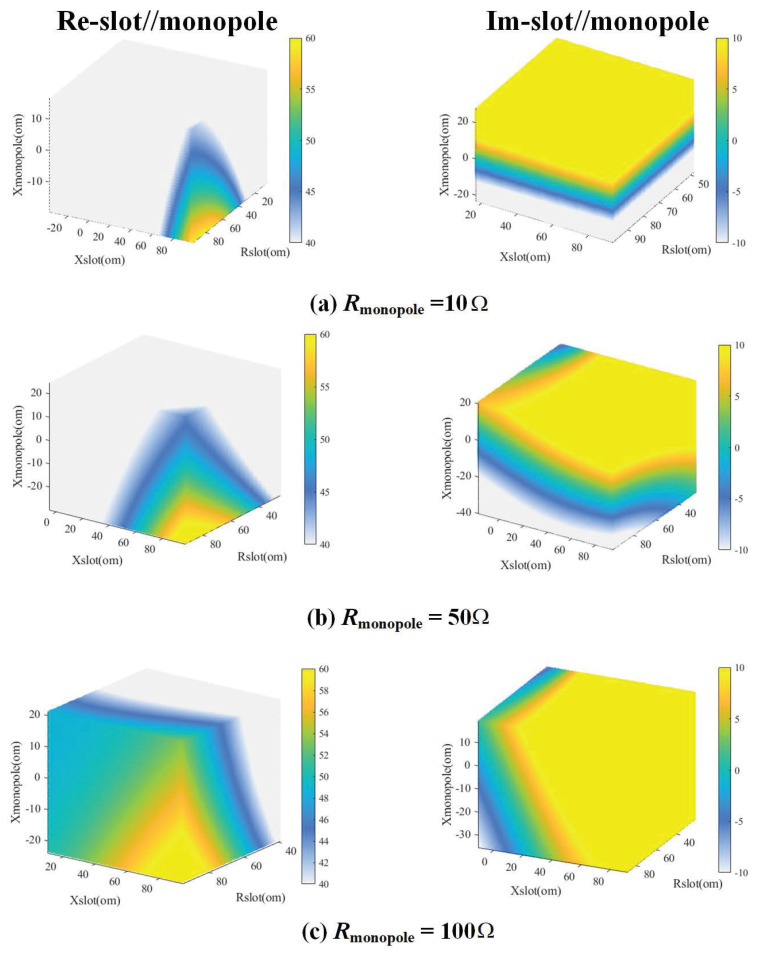
Calculated *Z*-parameters of the antenna element based on Equation (3) using MATLAB, when the real part of the impedance of the planar monopole is (**a**) 10 Ω, (**b**) 50 Ω, and (**c**) 100 Ω.

To further verify that the bandwidth can be enhanced by combining two antenna types in parallel, the four-dimensional image of calculated parallel impedance based on Equation (3) has been depicted in Figure 3. The coordinates of the four-dimensional image calculated by MATLAB are the real part and imaginary part of the impedance of the open-slot and the imaginary part of the impedance of the planar monopole, respectively. The fourth dimension of Figure 3 is the real part of the monopole. Then, the left column represents the real part of the parallel impedance, and the right is the imaginary part of it. Note that the right side of the image represents the colorbar. The colorbar of the real part extends from 40 Ω to 60 Ω, while the colorbar of the imaginary part ranges from −10 Ω to 10 Ω. It has been demonstrated that the optimum impedance match is achieved when the real part of the impedance is 50 Ω and the imaginary part is zero. Figure 3 shows that the color regions corresponding to the real part of 50 Ω and the imaginary part of 0 Ω have a continuous distribution and extensive coverage in the graph when the real part of the impedance of the planar monopole is 10 Ω, 50 Ω, and 100 Ω, which indicates that more resonant frequency points always exist whatever the real part and imaginary part of the open-slot antenna and planar monopole antenna vary in the case of parallel connection. Moreover, it can be confirmed that the bandwidth can be expanded by connecting the open-slot antenna and the planar monopole antenna in parallel.

**Figure 4:**
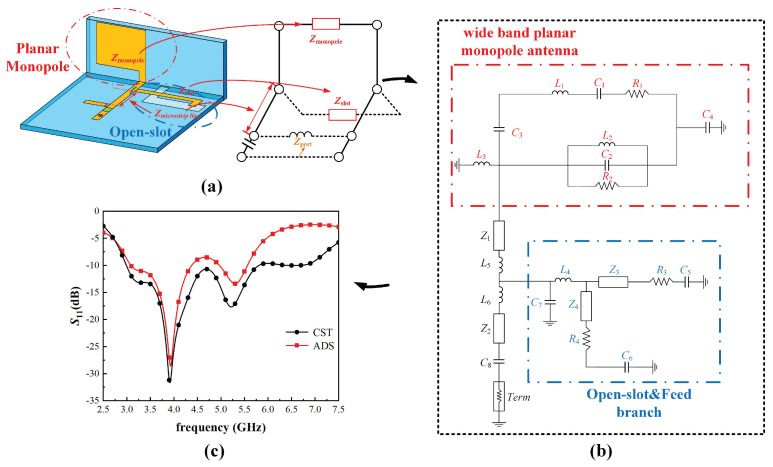
The equivalent circuit of the antenna element and simulated results: (**a**) The equivalent circuit of the antenna element; (**b**) The equivalent circuit established in ADS; (**c**) Simulated $S_{11}$ of the antenna element in CST and ADS.

Figure 4a shows the parallel equivalent model of the proposed antenna element based on Babinet’s principle. To verify the wideband principle of the MIMO antenna system, the equivalent circuit of the proposed antenna element made by ADS is established, as depicted in Figure 4. The circuits consisting of capacitors, resistors, and inductors represent wideband planar monopole antenna and open-slot and feed structure, respectively, and they are connected in parallel to form the equivalent circuit. *R*, *L*, *C*, *Z*, and *Term* represent the resistor, the inductor, the capacitor, the microstrip line, and the port termination with ground, respectively, and their detailed parameters and values in ADS, as illustrated in Table 1. Notice the microstrip substrate used in the microstrip line, which has a substrate thickness of 0.8 mm, a relative dielectric constant of 4.5, and a relative permeability of 1. Certain RLC circuits are utilized to express the impedance of the planar monopole, open-slot, and microstrip transmission line. The open slot is divided into two slots by a coupling-feed line, so $Z_{3}$ and $Z_{4}$ are deemed as the characteristic impedances of two slots, respectively [20,21,22], so it is deemed as a parallel circuit. The feeding branch is deemed as a parallel circuit consisting of $L_{4}$ and $C_{7}$ [20,23,24]. Then, the equivalent circuit of the open-slot and feed branch is established, which is circled in blue. The monopole is represented with a parallel or series RLC circuit [25,26,27]. Stored energy at the end of the monopole away from the ground plane is modeled with the inductance and capacitance [26]. So, the equivalent circuit of the wideband planar monopole antenna is also established, which is circled in red. Figure 4c demonstrates that the *S*-parameter curve simulated by CST Studio Suite is consistent with that of ADS, which indicates the accuracy of the equivalent circuit.

## 4. Prototype and Measured Results

**Figure 5:**
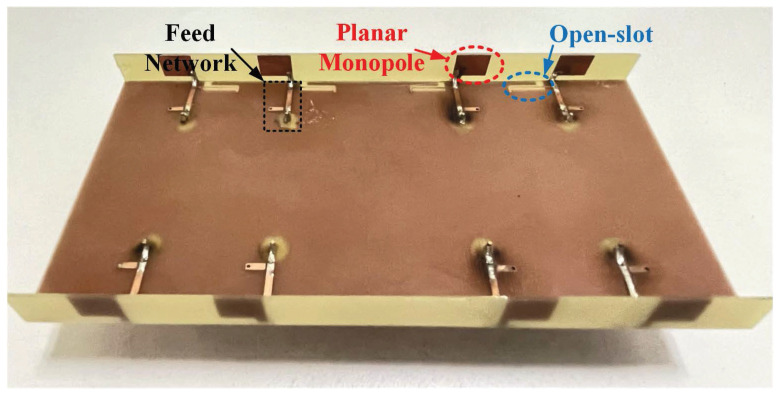
Prototype of the proposed MIMO antenna system.

To validate the performance of the proposed MIMO antenna system, a prototype was fabricated and measured, as shown in Figure 5. The simulated results were obtained using CST Studio Suite 2022. Moreover, the measurement system [28,29,30,31] used here is the General Test RayZone 1800, which operates at 0.4–8 GHz. It can measure many antenna characteristics such as bandwidth, 2D/3D radiation pattern, and efficiency. The rotary table used in the measurement system can achieve 360° rotation. The rotation accuracy of the rotary table is 0.5°. The maximum maximum speed is 30° per second. Eleven pairs of low RCS Vivaldi antennas of compact dimension were used in the measurement system to achieve the 15° RF sampling angular density defined in the CTIA measurement specification. With the addition of the horn antenna, there were 23 test antennas in total in this measurement system. By rotating the rotary table, all test antennas inside measured the data of each angle of the 2D radiation pattern. After rotating 180°, the 3D radiation pattern was obtained.

**Figure 6:**
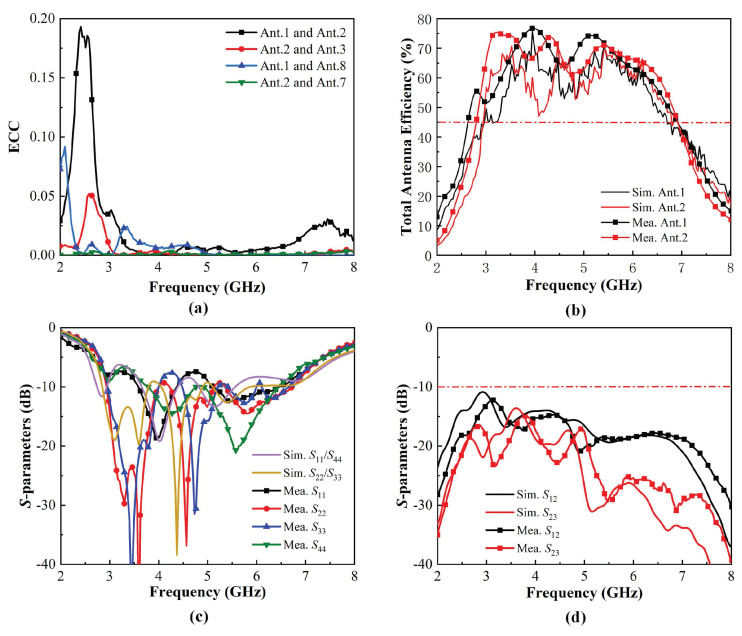
Simulated and measured results of the proposed antenna system: (**a**) Simulated and measured ECCs; (**b**) Simulated and measured total efficiencies; (**c**) Simulated and measured $S_{11}$, $S_{22}$, $S_{33}$ and $S_{44}$; (**d**) Simulated and measured $S_{12}$, $S_{23}$.

**Figure 7:**
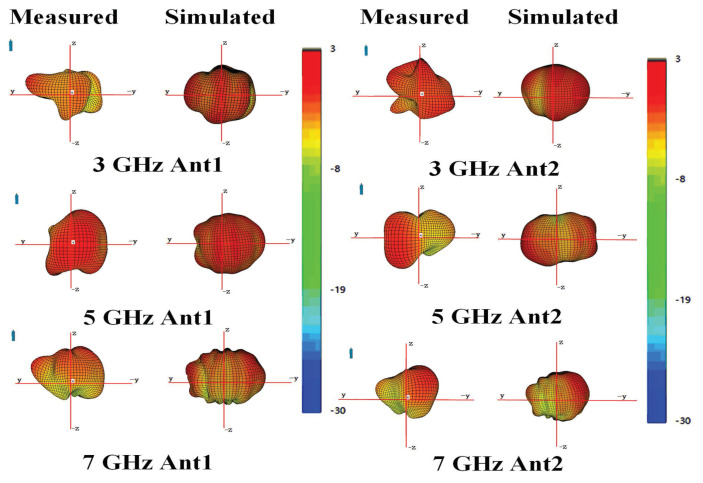
Simulated and measured 3D radiation patterns for the proposed antenna element.

Figure 6a,b illustrates the ECCs and total efficiencies. ECCs are lower than 0.05 within the bandwidth of 2.60–7.46 GHz. The measured total efficiencies of the proposed antenna element are higher than 45% within the operating band. The simulated and measured reflection coefficients and transmission coefficients are shown in Figure 6c,d. The 6-dB impendence bandwidths for reflection coefficients were obtained at 2.60–7.46 GHz. Isolation performance better than 12 dB was also obtained. Finally, Figure 7 shows the simulated and measured 3D radiation patterns of the proposed antenna. Results of Ant1 and Ant2 at three representative frequencies of 3, 5, and 7 GHz are presented. The inconsistency between Ant1 and Ant2 is caused by the different couplings around them. Errors exist due to darkroom equipment, but measured results agree with simulated results at gain extremes. Note that good agreement between all simulated and measured results was achieved, which indicates that the proposed MIMO antenna system has great potential for 5G and Wi-Fi 6E/7 mobile applications.

## 5. Conclusions

In this paper, a broadband MIMO antenna system was proposed for 5G and Wi-Fi 6E/7 terminal applications. First, the impedance properties of the open-slot antenna and planar monopole antenna were analyzed, connecting them in parallel to widen the proposed band. The four-dimensional image calculated using MATLAB based on Equation (3) further validated the feasibility of the proposed methodology. Then, based on impedance property analysis and Babinet’s principle, the broadband antenna element was designed in CST and the equivalent circuit of it was also established in ADS. The results simulated by CST are consistent with the results fitted by ADS, which indicates that the proposed methodology is effective. Finally, the wideband MIMO antenna system consisting of eight antenna elements was designed, fabricated, and measured. A 2.60–7.46 GHz bandwidth was achieved with isolation performance better than 12 dB. Acceptable antenna total efficiency higher than 45% was also obtained with ECCs lower than 0.05. Therefore, this paper provides a novel input impedance property analysis methodology based on Babinet’s principle to broaden bandwidth, which will be widely used in broadband antenna design.

## Figures and Tables

**Table 1:** Detailed component parameters and values in ADS.

Capacitive (pF)	$C_{1}$	$C_{2}$	$C_{3}$	$C_{4}$
	0.544	1.1278	1.6894834	1.84093
Capacitive (pF)	** $C_{5}$ **	** $C_{6}$ **	** $C_{7}$ **	** $C_{8}$ **
	3.500434	6.7033	0.7155926	1.5
Resistance (Ω)	** $R_{1}$ **	** $R_{2}$ **	** $R_{3}$ **	** $R_{4}$ **
	11.93	67.86	7.54	169.65
Inductance (nH)	** $L_{1}$ **	** $L_{2}$ **	** $L_{3}$ **	** $L_{4}$ **
	2.0755	1.07211	1.979	6.40966
Inductance (nH)	** $L_{5}$ **	** $L_{6}$ **		
	0.495	0.1		
	** $Z_{1}$ **	** $Z_{2}$ **	** $Z_{3}$ **	** $Z_{4}$ **
Width (mm)	1.4	1.4	0.5	0.7
Length (mm)	3.1	9.2	1	2.3
Impedance (Ω)	52	52	86	74

## Data Availability

The original contributions presented in the study are included in the article, further inquiries can be directed to the corresponding authors.
